# COVID-19 pandemic and antiretrovirals (ARV) availability in Nigeria: recommendations to prevent shortages

**DOI:** 10.11604/pamj.supp.2020.35.149.25639

**Published:** 2020-08-24

**Authors:** David Adelekan Dada, Emmanuel Aku, Kenneth Bitrus David

**Affiliations:** 1 Faculty of Pharmaceutical Sciences, Kaduna State University, Kaduna, Nigeria,; 2 Department of Microbiology, Kaduna State University, Kaduna, Nigeria

**Keywords:** COVID-19, HIV, antiretroviral, drug shortage

## Abstract

HIV/AIDS is an infectious disease that has claimed the lives of millions of people worldwide. Currently, there is no vaccine that has been developed in a bid to fight this deadly infection, however, antiretrovirals (ARVs), which are drugs used in the treatment of HIV infection are routinely prescribed to infected persons. They act via several mechanisms of action to reduce the severity of infection and rate of infectivity of the virus by decreasing the viral load while increasing CD4 counts. COVID-19 pandemic has resulted in unprecedented events affecting almost all areas of humans' life including availability of medicines and other consumables. This paper analyses the availability of ARVs during COVID-19 era and offered recommendations to be adopted in order to prevent shortages.

## Commentary

COVID-19 is an infectious disease that affects the respiratory tract and is caused by a novel strain of coronavirus called SARS-CoV-2. It was discovered in 2019 and has since become a global health crisis and was declared a pandemic on 11^th^March, 2020 by the World Health Organization (WHO) [1]. The emergence of COVID-19 has caused various sectors of the economy to experience great downfall and dwindles in its income generation and prosperity due to restriction of movement and business closure [2,3]. Antiretrovirals (ARVs) are medicines used for preventing retrovirus-Human Immunodeficiency Virus (HIV) from replicating. They are also known as HIV drugs. With Nigeria being the third largest country of people living with HIV/AIDS, the imminent economic shock especially on the health sector due to the COVID-19 pandemic has resulted in a decrease in the production capacity and distribution of ARV medicines leading to low availability of these medicines especially in third world country like Nigeria [4]. Analysis carried out by the UNAID on the potential impact of COVID-19 pandemic in low and middle income countries around the world on the supplies of generic ARV medicines used to treat HIV showed that the lockdown and closures imposed to curtail the spread of COVID-19 is greatly impacting the production of these medicines and as such could potentially lead to increase in the cost and shortage in the supply of these drugs [5]. The ripple effect of this anomaly indicts that from the 24.5 million people on ARV therapy, a great percent of that number are at increased risk of harm both to themselves and others owing to the increased risk of transmission because they cannot continue to gain access to their treatment.

Factors responsible for the low availability of ARV medicines across borders include: inadequate human resources in manufacturing facilities due to physical distancing and lockdown; curtailing of sea and air transport dents the manufacture and distribution of raw materials and other products such as packaging materials pharmaceutical companies needs to in order to manufacture these medicines. Other factors include: increased overhead and transport costs, the need for an alternative source of key starting materials and active pharmaceutical ingredients coupled with fluctuation and weakened national currency caused by envisaged economic shock apparently pushed up the cost of some antiretroviral regimens [6]. Additionally, the limited and reduced income due to COVID-19 shock at the level of the client, led to loss of follow-up and despite ARVs being free in third world countries like Nigeria for instance, clients still need to pay money for registration, laboratory and transportation amongst others and so they will tend to default in order to avoid this cost. Stern adherence to ARV therapy is compromised and resistance might develop due to loss of follow-up and stocks outs [6]. It is therefore expedient for countries to put in place the right mechanisms to enhance full stock of all antiretroviral drugs in order to avoid shortage as well as improve the drugs´ accessibility to the patients. A myriad of recommendations is being made by health experts, corporate bodies, civil societies and international health agencies to ensure sustainability of ARV supply chain amidst and beyond the COVID-19 pandemic.

Firstly, HIV therapy should be promptly integrated into social health insurance scheme if not in totality at least to a greater percentage at all tiers of government such that a pool of funds is created thus, enhancing domestic resource mobilization without further pushing people into poverty. A coordinated action by government and private organizations is necessary for even distribution of ARVs to persons who need HIV treatment [7]. Secondly, the ongoing sustainability efforts such as the HIV Trust Fund should not be abandoned. Intensified efforts should be made to encourage private sectors to continually be supportive despite the prevailing circumstances. Political leaders and lawmakers should ensure that intensified efforts for continual public funding of HIV treatment is not overlooked [7]. Lastly, countries around the world especially those greatly hit by the COVID-19 pandemic should use this opportunity to increase efforts and commitments to building a vibrant health system with an expanded health workforce. A robust health system will help enhance the benefits of economies of scale which are required to effectively respond to ongoing and emerging global health crises such as what is seen in the COVID-19 pandemic [7].

## Conclusion

The economic impact of COVID-19 pandemic indicates a possible shortfall in the availability and a rise in the cost of ARVs. We cannot let the COVID-19 pandemic undo the hard-won gain in the global response to this disease. In different occasion the executive director UNAIDS Winnie Byanyama said: '*it is vital that countries urgently make plans now to mitigate the possibility and impact of higher cost and reduced availability of antiretroviral medicines*'. It is a call for global action by both suppliers and buyers to ensure smooth delivery and accessibility of ARV medicine for continue treatment by person living with HIV. Government should also pull concerted efforts and create policies that will enable this set of people gain uninterrupted access to antiretroviral in the midst of the current pandemic and beyond this global health crisis.
